# The future of successful aging in Alaska

**DOI:** 10.3402/ijch.v72i0.21186

**Published:** 2013-08-05

**Authors:** Jordan Lewis

**Affiliations:** Department of Psychiatry and Behavioral Sciences, University of Washington School of Medicine, Seattle, WA, USA

**Keywords:** Alaska Native, older adults, successful aging, intergenerational, rural

## Abstract

**Background:**

There is a paucity of research on Alaska Natives and their views on whether or not they believe they will age successfully in their home and community. There is limited understanding of aging experiences across generations.

**Objective:**

This research explores the concept of successful aging from an urban Alaska Native perspective and explores whether or not they believe they will achieve a healthy older age.

**Design:**

A cultural consensus model (CCM) approach was used to gain a sense of the cultural understandings of aging among young Alaska Natives aged 50 years and younger.

**Results:**

Research findings indicate that aging successfully is making the conscious decision to live a clean and healthy life, abstaining from drugs and alcohol, but some of Alaska Natives do not feel they will age well due to lifestyle factors. Alaska Natives see the inability to age well as primarily due to the decrease in physical activity, lack of availability of subsistence foods and activities, and the difficulty of living a balanced life in urban settings.

**Conclusions:**

This research seeks to inform future studies on successful aging that incorporates the experiences and wisdom of Alaska Natives in hopes of developing an awareness of the importance of practicing a healthy lifestyle and developing guidelines to assist others to age well.

The state of Alaska is facing an increasing growing senior population among Alaska Natives and non-Natives and because of this it will be important for us to better understand the benefits and challenges of growing older in Alaska. The number of Alaska Native seniors aged 60 and above continues to increase rapidly. According to the Alaska Commission on Aging, from the actual 2010 census to 2011 projected population data, the Alaska Native senior population increased 8% statewide. The highest regional growth rate over this period was in the Aleutians (121.3%) followed by south-central Alaska (117.2%) (1). In addition to a growing elderly population, the demographic realities and shifts in Alaska with the rapid outmigration of younger family members from rural to urban communities, and escalating prices and cost of living in rural Alaska, are having direct impacts on changing notions of successful aging for Alaska Natives. As rural communities shift, the experiences and needs of our families will change, as well as the traditional roles of our Elders.

In addition to living longer, many older adults are living with multiple chronic illnesses, such as diabetes and heart disease. The presence of chronic conditions has become epidemic. In the United States, more than 133 million people, or nearly half of the population, suffer from a chronic condition (2). Among our Alaska Native youth, we have high rates of suicide, accidental deaths and other factors contributing to shorter lifespan (3). Alcohol abuse has devastating health and social consequences for many Alaska Natives. Cirrhosis death rates for Alaska Natives were 18.7 per 100,000 in contrast to the US baseline of 9.6, and alcohol is linked to 72% of suicides among Alaska Native males aged 15–24 – a group with a suicide rate 14 times the national rate (3,4). Because of these escalating rates of multiple chronic illnesses and growing population in Alaska, it is important for Alaska Natives to better understand what is needed to ensure a successful older age because it will enable them to get the most out of their lives, feel good about themselves, as well as preserve their culture and traditions, while honoring the Elders who share their knowledge and wisdom on healthy living. Rowe and Kahn's (5) biomedical definition of successful aging emphasizes physical health and absence of disease, high cognitive and physical functioning, and active engagement with life and serves as the foundation of this article. The findings of this article fit within this model, but focuses on the factors that undermine the ability of Alaska Natives to age well or follow the advice of their Native Elders on how to age well.

Very little research has been conducted with younger generations of Alaska Natives to explore whether or not they believe they will age well and why or why not. This study hopes to address this gap in the literature and serves as an example of why we need to explore healthy aging among our youth to develop guidelines to ensure a long and healthy life. Baltes and Smith (6) make the argument that research needs to shift its focus to the entire spectrum of human development, avoiding a depletion of resources for all age groups. They state: “Perhaps the time has come to think about the younger ages in order to serve old age” (6, p. 124), which supports the focus of this article.

## Literature

One of the challenges of conducting research on successful aging with cultural groups (e.g. Alaska Natives) is the lack of data, or research, on culture and aging, and its impact on how we view successful aging. There have been few studies of successful aging that have considered what role culture plays in this construct. Clark and Anderson (7) were among the first to recognize that the realities of minority elders had been neglected when sorting out successful and unsuccessful agers. Some studies have attempted to understand what successful aging means in different cultures (8–13), but challenges still exist.

### Definition of successful aging

Rowe and Kahn's (5) definition of successful aging is based on the biomedical model of aging, focusing on 3 components: low probability of disease and disease-related disability, high cognitive and physical functional capacity, and an active engagement with life. This definition, which has served as the basis of other research on successful aging, was criticized as being more focused on health and physical functioning and less inclusive on the individual and their environment (14). Another major criticism of the existing successful aging literature has been the failure to take into account the perspective of those being studied about what defines and determines successful aging (15), which can be applied to the thoughts and experiences of the younger generation and their perspectives on growing older.

According to Phelan et al. (16), the validity of successful aging and an empirical understanding of its utility and relevance could be enhanced if beliefs of the general public were obtained and incorporated into researchers’ definitions. Asking individuals about the meaning and relevance of successful aging would enrich the theoretical definitions that have dominated this area of research. While it seems clear that gerontologists remain unclear about the definition of successful aging, the term appears to have personal meaning for elderly people, which may also be true for all ages. In one study, only 2% of older persons could not define successful aging (17), suggesting that elders have particular perspectives on what aging well means for them. An indigenous-centred definition is essential for future research in the fields of successful aging and community health, for it would more appropriately inform understandings about successful aging within a particular population (16).

## Design and methods

A purposive sampling procedure was used in this study to interview 7 individuals (N=7) who were not considered Elders by their respective community; participants were under the age of 50. Prior to conducting the interviews, IRB human subject protection was obtained through the University of Alaska Fairbanks (UAF) Office of Research Integrity to ensure protection of the participants.

Among the participants, 4 Alaska Native tribal groups (Gwich'in Athabascan, Inupiaq, Tlingit and Yup'ik Eskimo) were represented who were residents of 2 different urban communities and self-identified as Alaska Native. There were 4 women and 3 men and they all live in an urban community in Alaska. For the purposes of this study, urban communities are defined as Anchorage or Fairbanks. The age range was 26–50 and 100% of the participants were either full-time employed or pursuing a higher education at the University of Alaska.

The study used the cultural consensus model (CCM) to explore whether there was agreement on the concept of successful aging among Alaska Native cultural groups. Dressler (18) states that the “degree of consensus on a model and the degree to which individuals share in that consensus can be estimated quantitatively using the cultural consensus model” (p. 27). He argues that working from the pattern of agreement regarding some domain, in this case successful aging, using a relatively small population size, this model provides a reasonable inference that the individuals share a model of that domain. Romney et al. (19) believe that culture is most usefully studied in terms of knowledge and that the most important characteristic of cultural knowledge is that it is shared among those being studied. For this study, the CCM asked 20 questions covering topics, such as how Alaska Natives define successful aging, how their aging process affects their emotional, spiritual and cognitive well-being. This study offers suggestions of successful aging grounded in the experiences and perceptions of Alaska Natives, highlighting the challenges faced by the younger generations.

### Data collection

The one-on-one interviews consisted of open- and close-ended questions, allowing time to gather demographic information as well as to ask open-ended questions that enabled the participants to share stories and elaborate on their answers. All the interviews were conducted over the phone and hand-written notes were kept that outlined the responses to the interview questions. Once the interviews were completed and notes were typed up for each person, they were sent back to the participants for review and corrections if needed. Each participant received a small token of appreciation for his/her time participating in the study.

### Data analysis

A grounded theory approach (20) was used to gain a sense of the perspectives of aging in Alaska. With the use of this methodology, there is a continual interplay between data collection, data analysis and theory development. Theory development occurs in the midst of data collection, rather than taking place after the data collection, and those who use grounded theory often do qualitative research by making direct observations or conducting interviews in field settings. Strauss and Corbin (21) define grounded theory as a theory that is derived from data, systematically gathered and analyzed throughout the research process.

This approach to data analysis allowed the author to determine common themes expressed in the experiences and stories. The lived experiences of the participants were most important and not the researcher's understanding, or interpretation, of successful aging. Rather than bringing ideas and thoughts to the study, this article provides insight on successful aging among the participants, using their words.

## Results

This study looked at the concept of successful aging among the younger generations (age: 50 and younger) to gain a sense of how the successful aging is defined and determine whether or not they believe they will achieve a healthier old age. This section highlights their views and beliefs on healthy aging.

The participants defined successful aging as being psychologically and physically comfortable and having a support system. Based on the interviews, their definition of successful aging was more aligned with Rowe and Kahn's biomedical definition (5). One individual stated that aging well to her meant “maintaining good physical and mental health, facing challenges associated to aging with courage and humility.” A Yup'ik woman stated that her health was important and the only way to ensure she would age well is “keeping physically busy and eating well (Yup'ik foods).”

## Lifestyle

Most of the participants grew up in rural villages but currently reside in urban communities where they are employed or attend the university. Similar in nature to other urban settings, many of the jobs in Alaska require sitting behind a desk, preventing them from engaging in subsistence activities or being physically active. One of the interesting findings in the interviews was their fear of not aging successfully because of their employment and not being able to engage in physical activities. The main reason they are unable to engage in physical activities, or be on the land, is because they are stuck in the office behind their computers. An Inupiat woman stated: “I am trying to keep busy. It's hard when you have a job and sit all day.” Most stated that people were much busier long ago because they didn't have televisions and computers. “People just sit there now. There are not many activities where men go out each season to subsist,” was how a participant illustrated the differences. In relation to the sedentary lifestyle and being stuck in an office, a young Yup'ik woman stated that she “tries to keep busy. I go for a walk every once in a while to get exercise and I try to eat well.”

Some of the respondents focused on physical activity and maintaining a healthy lifestyle when asked how they define and try to age well. They primarily focused on a holistic approach to health (physical, mental, spiritual), which is common among many indigenous views of health and well-being. Some of the participants included mental well-being and accepting the fact they are going to be growing older, but they are concerned about what they should do to ensure they would age successfully. When asked how he tries to age well, a Tlingit man gave a holistic approach to his health. He stated: “I live a balanced life without alcohol and drugs. I take care of myself and consciously eat healthy foods regularly, exercise, don't drink or use drugs. Live spiritually.” One Aleut lady stated that she has tried to age well by “by mentally accepting that aging is a healthy, natural, transition. Responsibilities change, but it's still rewarding.”

## Rural versus urban lifestyles

Differences in how someone ages in a rural versus an urban community emerged during the interviews. Most of the participants stated that people were much busier long ago because they did not have televisions and computers and spent a majority of their time on the land to provide for their families and communities. Remaining active was critical to their survival, but also instrumental in maintaining good physical and mental health, which contributed to a sense of well-being and having a positive outlook on life.

A Gwich'in Athabascan man noted a difference between rural and urban lifestyles. “There is a huge difference. A physical difference. Rural communities have more physical activities, for example, hauling water. They don't haul water in the cities.” He suggested that the question should be restated; instead of dichotomizing rural vs. urban, the question should be rephrased as “traditional way of living vs urban living.” He goes on to state that:

Rural is a Western concept and focuses less on a Native way of living. In urban communities, food isn't traditional, rural has more traditional food, which is like our medicine. Living off of the land helps people age well; food connects who we are as Native people. Living off the land is putting your body to use. Keeping up your health and mental balance. Don't have these opportunities in the cities.

As rural communities continue to make advances with technology, and Elders pass on, the role of traditional leaders and knowledge bearers will fall to our youth and we need to ensure they live a healthy and productive life and pass on the culture and traditions.

## Knowledge of successful aging

In addition to asking how the participants have tried to live a healthy life, another question asked where they received most of their information on what it means to age successfully. Most of the respondents indicated that they observed and followed in the steps of immediate family members and relatives. An Inupiaq woman stated: “I got most of my information about aging well from family members who are elderly.” The Yup'ik woman stated: “I get most of my information about aging well by observing.” An Aleut woman sums it up by saying “thinking about those before me who aged. I read some things, but I think of my parents and grandparents as models.” Almost every respondent had a role model(s) to follow who exemplified successful aging and how to live their lives to the fullest.

## What is poor, or unhealthy, aging?

Two Alaska Native women explained they would age poorly if they engaged in self-abuse and did not take care of themselves. One Inupiat woman defined the symptoms of poor aging as, “Self-abuse and self-sabotage. You don't take care of other people, particularly older people.” Some of the respondents emphasized the importance of helping others and giving back to the community. One Gwich'in Athabascan man stated that he has tried to age well through “working with the community and the youth. Helping with the problems of the community. I don't just talk about the problems, I work on them.”

The participants also placed emphasis on biological health and genetics. A Yup'ik woman stated that you need “good genes” to age successfully. When asked how the participants have tried to age well, a majority of the participants placed more emphasis on remaining active and being free of disease, which supports Rowe and Kahn's (5) biomedical definition of successful aging.

## Conclusion

Acquiring a better understanding of successful aging could be enhanced if beliefs and definitions were elicited from the public and incorporated into researchers’ definitions. Asking individuals about the relevance and meaning of successful aging will enrich the definitions, making them more applicable to the diverse aging population. The 3 key observations of this study are as follows. First, the current lifestyles of the participants are preventing them from engaging in activities to stay active, which they believe will enable them to age well like the Elders and family members before them. This observation is supported by activity theory (22,23), which states that staying active is an important component to successful aging, and inactivity results in poor aging, which is the fear of the participants in this study. Second, participants focused on their physical and mental health and differences in technology use. Reichstadt et al. (15) stated that a majority of the studies on successful aging cited physical health as a critical factor to successful aging, which was found to be an important aspect of aging for this study. Duay and Bryan (24) also found health to be a critical component of successful aging. They found that, “health is an extremely important issue with this group of participants. Over 70% of them mentioned health or physical activity when asked to describe someone they know who is aging successfully or provide advice to younger people on aging successfully” (24, p. 434). Third, and final, the demographic realities and shifts in Alaska with the rapid outmigration of younger family members from rural to urban communities, and escalating prices and cost of living, impacts on changing notions of successful aging for Alaska Natives. Based on these observations, we need to develop guidelines for healthy living in later life to ensure they become healthy Elders.

Based on the results of the interviews with the participants, they all have a desire to live a healthy and balanced life, but the demands of living in urban settings and working to provide for their families prevents them from activities they believe will help them age well. Blazer (25) summarized the importance of learning from others on how to live a healthy life in his editorial: “We have the privilege of learning from our parents, our patients, and our mentors the true meaning of successful aging” (25, p. 5). Each participant understood the value and importance of aging well and looked up to family and community members who served as examples, but found it challenging to engage in the same activities and behaviors because of constraints and demands in their current work and home environment. The findings in this study lead to future research to explore how Alaska Natives living in urban settings maintain a connection to their natal community and continue traditional, or cultural, practices. As more Alaska Natives relocate to urban communities for various reasons, it will be important to determine how they maintain cultural connections, access to their Native foods and maintain family and community connections. Future research will need to explore the notion of urban successful aging among Alaska Natives, how they can maintain a connection to their culture and identify new successful aging practices for this population. A larger sample size of Alaska Natives with broader demographics would provide data that would be much more meaningful and give deeper insight into the challenges of successful aging in urban Alaska.

Insight into how successful aging is defined by Alaska Natives will inform the factors that determine whether or not communities are able to meet their needs and enable them to achieve a healthy old age.

## References

[1] Senior snapshot: older Alaskans in 2011/2012 http://www.alaskaaging.org.

[2] Partnership for solutions: chronic conditions: making the case for ongoing care http://www.rwjf.org/programareas/resources/product.jsp?id=14685&pid=1142&gsa=pa1142.

[3] Allen JA, Mohatt GV, Rasmus SM, Hazel KL, Thomas L, Lindley S (2006). The tools to understand: community as co-researcher on culture-specific protective factors for Alaska Natives. J Prev Interv Community.

[4] Alaska Department of Health & Social Services [DHSS] (2002). Healthy Alaskans 2010: targets and strategies for improved health.

[5] Rowe JW, Kahn RL (1987). Human aging: usual and successful. Science.

[6] Baltes PB, Smith J (2003). New frontiers in the future of aging: from successful aging of the young old to the dilemmas of the fourth age. Gerontology.

[7] Clark M, Anderson BG (1967). Culture and aging: an anthropological study of older adults.

[8] Ikels C, Dickerson-Putnam J, Draper P, Cutler SJ, Bond LA, Grams A (1995). Comparative perspectives. Promoting successful and productive aging.

[9] Keith J, Fry CL, Ikels C, Sokolovsky J (1990). Community as context for successful aging. The cultural context of aging.

[10] Keith J, Fry CL, Glasock AP (1994). The aging experience: diversity and community across cultures.

[11] Lewis J (2011). Successful aging through the eyes of Alaska Natives. What it means to be an elder in Bristol Bay, AK. Gerontologist.

[12] Tate RB, Leedine L, Cuddy TE (2003). Definition of successful aging by elderly Canadian males: the Manitoba follow-up study. Gerontologist.

[13] Torres S (2006). Different ways of understanding the construct of successful aging: Iranian immigrants speak about what aging well means to them. J CrossCult Gerontol.

[14] Strawbridge WJ, Wallhagen MI, Cohen RD (2002). Successful aging and wellbeing: self-rated compared with Rowe & Kahn. Gerontologist.

[15] Reichstadt J, Depp CA, Palinkas LA, Folsom DP, Jeste DV (2007). Building blocks of successful aging: a focus group study of older adults’ perceived contributors to successful aging. Am J Geriatr Psychiatry.

[16] Phelan EA, Anderson LA, LaCroix AZ, Larson EB (2004). Older adults’ views of ‘successful aging’ – How do they compare with researchers’ definitions?. J Am Geriatr Soc.

[17] Fisher BJ (1995). Successful aging, life satisfaction, and generativity in later life. Int J Aging Hum Dev.

[18] Dressler WW (2004). Culture and the risk of disease. Br Med Bull.

[19] Romney AK, Weller SC, Batchelder WH (1986). Culture as consensus: a theory of culture and informant accuracy. Am Anthropol.

[20] Glaser BG, Strauss AL (2006). The discovery of grounded theory: strategies for qualitative research.

[21] Strauss A, Corbin J (1998). Basics of qualitative research. Techniques and procedures for developing grounded theory.

[22] Lemon BW, Begtson VL, Peterson JA (1972). An exploration of the activity theory of aging: activity types and life satisfaction among in-movers to a retirement community. J Gerontol Soc Sci.

[23] Menec VH (2003). The relation between everyday activities and successful aging: a 6-year longitudinal study. J Gerontol Soc Sci.

[24] Duay DL, Bryan VC (2006). Senior adults’ perceptions of successful aging. Educ Gerontol.

[25] Blazer DG (2006). Successful aging. Editorial. Am J Geriatr Psychiatr.

